# Rodent group borreliae do occur in wild rodents from the Caribbean region of Colombia

**DOI:** 10.1186/s13071-024-06560-7

**Published:** 2024-11-29

**Authors:** Yesica López, Ketty Galeano, Álvaro A. Faccini-Martínez, Sebastián Muñoz-Leal, Yeimi Lopez-Mejia, Marina Muñoz, Juan D. Ramírez, Camilo Guzman, Alfonso Calderon, Salim Mattar

**Affiliations:** 1 instituto de Investigaciones Biológicas del Trópico, Universidad de Córdoba, Córdoba, Colombia; 2 Servicio de Infectología, Hospital Militar Central, Bogotá, Colombia; 3 Facultad de Medicina, Universidad Militar Nueva Granada, Bogotá, Colombia; 4 Departamento de Ciencia Animal, Facultad de Ciencias Veterinarias, Universidad de Concepción, Chillán, Chile; 5 Instituto de Biotecnología – UN (IBUN), Universidad Nacional de Colombia, Bogotá, Colombia; 6 Centro de Investigaciones en Microbiología y Biotecnología-UR (CIMBIUR), Facultad de Ciencias Naturales, Universidad del Rosario, Bogotá, Colombia; 7 Molecular Microbiology Laboratory, Department of Pathology, Molecular and Cell-Based Medicine,, Icahn School of Medicine at Mount Sinai, New York City, NY USA

**Keywords:** *Borrelia*, *Zygodontomys* sp., Rodent reservoir hosts, Zoonosis, Tick-borne diseases

## Abstract

**Background:**

Bacteria of the genus *Borrelia* are agents of disease in both domestic animals and humans and pose a significant public health risk. *Borrelia* species have complex transmission cycles, often using rodents as vertebrate reservoir hosts. These bacteria are classified into three well-defined monophyletic groups: *Borrelia burgdorferi* sensu lato (Bbsl) complex, the relapsing fever (RF) group, and a third group associated with reptiles and echidnas. Moreover, a new group of *Borrelia* associated with rodents has recently been proposed, as these bacteria form a phylogenetic group separated from the previously mentioned groups. This study aimed to investigate the presence of DNA of *Borrelia* spirochetes in rodents in specific areas of the Colombian Caribbean.

**Methods:**

A total of 155 rodent spleen samples were selected from the tissue bank. These samples were obtained in the departments of La Guajira and Córdoba (Northern Colombia). DNA extraction and specific real-time polymerase chain reaction (PCR) targeting Borrelia 16S ribosomal RNA (rRNA) gene were performed, followed by nested PCR (nPCR) on positive samples to obtain larger fragments of the 16S rRNA gene and characterize the *flaB* gene. Alignments of generated sequences and ortholog sequences downloaded from Genbank were performed in Clustal Omega. A phylogenetic tree was built with the maximum likelihood method in IQTREE.

**Results:**

Spleen samples from rodents of the genera *Heteromys*, *Mus*, *Necromys*, *Olygoryzomys*, *Proechymis*, *Rattus*, *Sigmodon*, and *Zygodontomys* were processed. Overall, 6.5% (4/162) of the animals tested positive for *Borrelia* by real-time PCR. All quantitative PCR (qPCR)-positive samples were also positive for nPCR targeting the 16S rRNA gene, yielding fragments of 344–408 bp and 603–673 bp from two *Sigmodon* rodents and two *Zygodontomys* rodents from La Guajira and Córdoba. All samples were negative for the *flaB* gene. Only samples from *Zygodontomys* rodents presented good quality sequences. A BLASTn analysis showed a percentage of identity ranging between 98.16 and 96.06% with *Borrelia* sp. R57. Phylogenetic analysis revealed that sequences of the present study clustered with species of the recently proposed *Borrelia* “rodent group.”

**Conclusions:**

This is the first detection of borreliae of the “rodent group” in South America. Our results reaffirm the occurrence of a group of spirochetes associated with rodents, extending its geographic distribution to the Colombian Caribbean.

**Graphical abstract:**

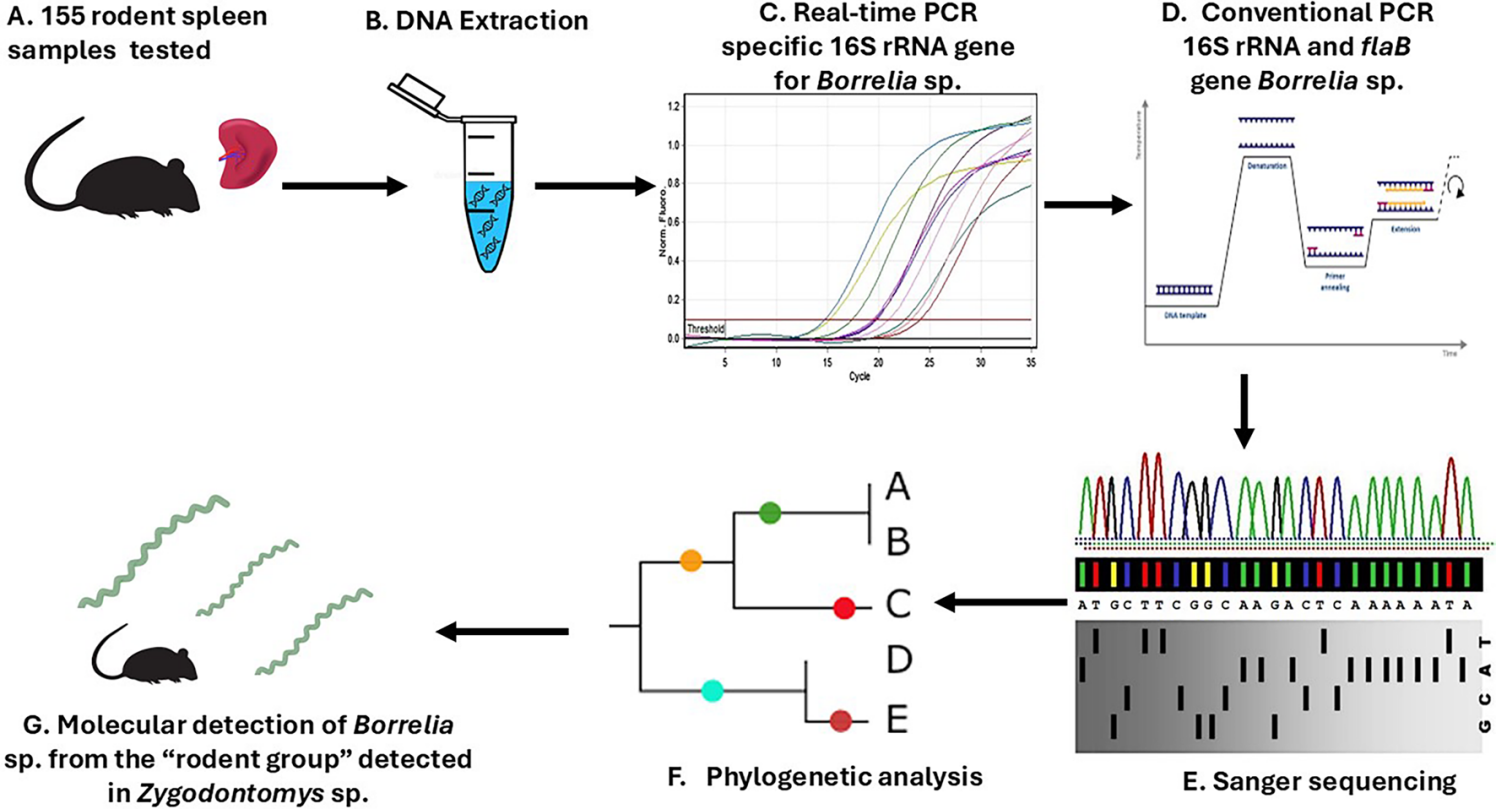

**Supplementary Information:**

The online version contains supplementary material available at 10.1186/s13071-024-06560-7.

## Background

The genus *Borrelia* includes pathogenic species that cause emerging and reemerging zoonotic diseases of significance to human and animal health [1]. The genus *Borrelia* is composed of three main monophyletic groups: the *Borrelia burgdorferi* sensu lato group (Bbsl), the relapsing fever (RF) group, and a group associated with reptiles and echidna (*Tachyglossus aculeatus*) hosts [2, 3]. Generally, borreliae are transmitted by ixodid (Ixodidae) and argasid (Argasidae) ticks, and one species, *Borrelia recurrentis*, is transmitted by the human clothing louse (*Pediculus humanus humanus*) [3]. Additionally, vertebrate hosts, such as bats, armadillos, monkeys, opossums, wild turkeys, deer, and squirrels, may be involved in the transmission cycle [1, 4–7]. But rodents are one of the most important vertebrate hosts for *Borrelia* spp. [8–10].

Indeed, many studies support this fact. In 1927, it was determined that the hosts of *Borrelia* of the RF group were several species of rodents [11]. Furthermore, in 1989 in the USA, *B*. *burgdorferi* was isolated from the rodent *Peromyscus leucopus*, which was at that time the main reservoir [12]. Subsequently, in the 1990s, *Borrelia* spp. of the Bbsl group were isolated in Europe in four species of rodents [13]. Furthermore, the rodent *Oryzomys palustris* is also a reservoir host for *B*. *burgdorferi* [14]. In South America, Thomas et al. shed light on the role of rodents as possible reservoirs of *Borrelia* spp. by detecting species of the Bbsl and RF group in rodents from Chile [15]. Moreover, a recent detection of those two groups of *Borrelia* spirochetes in rodents from Colombia, reinforces this association [16].

However, recent findings point that rodents carry spirochetes of genus *Borrelia* that form a consistent group that separates from Bbsl and RF groups from a phylogenetic viewpoint. In fact, a new group of borreliae associated with rodents in Australia, Spain, and the USA has been proposed and named as the “rodent group” [17–19], and adds a fresh perspective to the diversity of the genus [17]. In this study, we had access to a large number of rodent organs collected in the Colombian Caribbean and aimed to detect *Borrelia* DNA.

## Methods

### Study area and capture of rodents

**Fig. 1:**
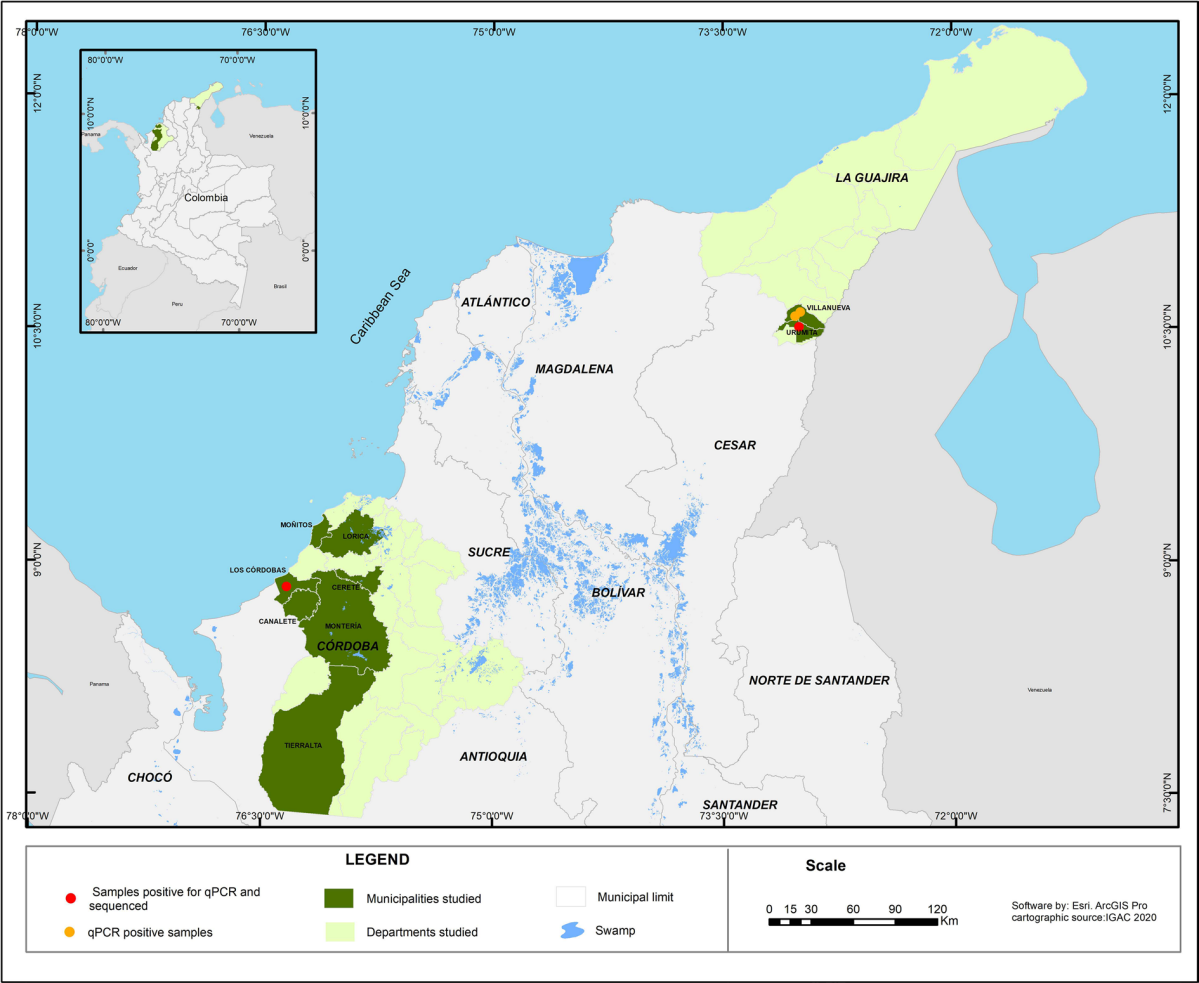
Map of Colombia showing the location of the municipalities sampled in the departments of Córdoba and La Guajira. The points indicate the collection sites of qPCR-positive rodents and the sequenced samples

Spleen samples of rodents were obtained from the tissue bank of the Instituto de Investigaciones Biológicas del Trópico (Universidad de Córdoba). Rodent species from which spleen samples were collected were previously captured in field trips between 2011 and 2012 and 2022 and 2023 in rural and peri-urban areas of the municipalities of Los Córdobas, Montería, Tierralta, Moñitos, Cereté, and Lorica in the department of Córdoba; and rural areas of the municipalities of Villanueva and Urumita from the department of La Guajira (Additional file 1: Table S1) (Fig. 1).

### Molecular and phylogenetic analyses

DNA extraction from spleen was performed using the GeneJET genomic DNA purification kit (Thermo Scientific) following the manufacturer’s instructions. The DNA was quantified with a spectrophotometer. As an internal control, conventional polymerase chain reaction (PCR) targeting the mammalian *β-actin* gene was performed [20]. Real-time PCR (qPCR) was performed to detect *Borrelia* spp. using genus-specific primers (Bor16S3F, 5′-AGC CTT TAA AGC TTC GCT TGT AG-3′; Bor16S3R, 5′-GCC TCC CGT AGG AGT CTG G-3′) and a hydrolysis probe (Bor16S3P, 5′-6FAM–CCG GCC TGA GAG GGT GAA CGG BHQ-3′) that amplify a 148 bp fragment of the 16S ribosomal RNA (rRNA) gene [21]. Nested PCR (nPCR) and semi-nested PCR were implemented to positive samples to amplify three overlapping fragments of the 16S rRNA gene and two overlapping fragments of the *flaB* gene (Table 1) [22, 23].

**Table 1:** Nested PCR primers for the detection of *Borrelia* sp.

Gene	Round	Primer name	Secuencia 5′–3′	Tm [°C]	bp
16S-rRNA [22]	First round	FD3 [f]	AGAGTTTGATCCTGGCTTAG	54	1489
		T50 [r]	GTTACGACTTCACCCTCCT		
	Second round (hemi nested A)	FD3 [f]	AGAGTTTGATCCTGGCTTAG	56	730
		16 s-1[r]	TAGAAGTTCGCCTTCGCCTCTG		
	Second round (hemi nested B)	16 s-2 [f]	TACAGGTGCTGCATGGTTGTCG	56	462
		T50 [r]	GTTACGACTTCACCCTCCT		
	Second round (nested)	Rec4 [f]	ATGCTAGAAACTGCATGA	54	520
		Rec9 [r]	TCGTCTGAGTCCCCATCT		
*flaB* (flagellin*)* [23]	First round	FlaRL[f]	GCAATCATAGCCATTGCAGATTGT	55	665
		FlaLL[r]	ACATATTCAGATGCAGACAGAGGT		
	Second round A	FLaRS [f]	CTTTGATCACTTATCATTCTAATAGC	55	491
		FlaLL[r]	ACATATTCAGATGCAGACAGAGGT		
	Second round B	FlaRL[f]	GCAATCATAGCCATTGCAGATTGT	55	528
		FLaLS [r]	AACAGCTGAAGAGCTTGGAATG		

*Borrelia anserina* genomic DNA was used as a positive control [24] and molecular grade water was used as a negative control. Sanger sequencing was performed on nPCR-positive samples. Sequences with a Phred score of 20 in Geneious (https://www.geneious.com/) were chosen and a taxonomic assessment of the sequences was performed with BLASTn [25], considering an E-value ≤ 1 × 10^−5^, a high Max Score and Total Score, a Query Cover > 90%, and a Per. Ident > 90%.

Sequences from the distinct groups of the *Borrelia* genus were downloaded from Genbank [26], to align them with the sequences of this study. Alignments were performed in Clustal Omega [27]. Phylogenetic reconstructions were performed in IQTREE with the maximum likelihood method using the TPM3 + F + G4 chosen according to BIC (Bayesian Information Criterion). We used a nucleotide substitution model with 1000 bootstraps [28]. Trees were visualized and edited with iTOL v5 [29].

## Results

In total, 155 spleen samples from eight genera of rodents were analyzed; 42.6% (66/155) were of genus *Rattus*, 31.6% (49/155) *Zygodontomys*, 10.9% (17/155) *Olygoryzomys*, 5.1% (8/155) *Mus*, 3.9% (6/155) *Proechymis*, 1.9% (3/155) *Heteromys*, 1.9% (3/155) *Sigmodon*, and 1.9% (3/155) *Necromys*.

**Fig. 2:**
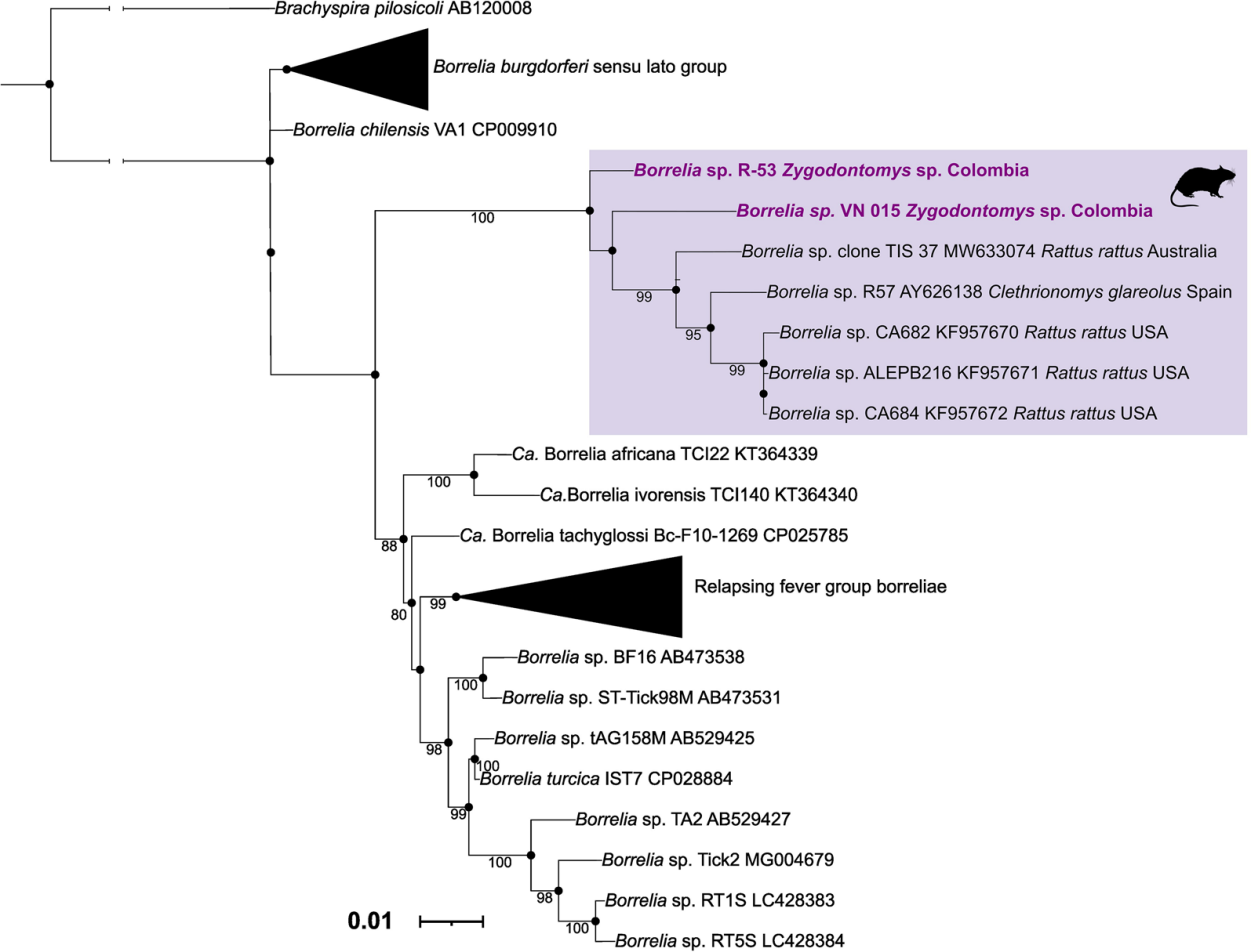
Phylogenetic analysis performed in this study. Phylogenetic tree of 16S rRNA gene constructed with 72 sequences, including two own sequences obtained in this study. The phylogenetic clade related to rodents is colored purple; the position of the detected *Borrelia* spp. is highlighted in bold purple. *Brachyspira pilosicoli* was used as an external group. The tree was built using the nucleotide substitution model TPM3 + F + G4 chosen according to BIC. The tree is drawn to scale, with the scale bar indicating nucleotide substitutions per site

All samples were positive for the *ß-actin* gene PCR, demonstrating successful DNA extractions. The 6.5% (4/162), two *Zygodontomys* and two *Sigmodon* specimens, were positive for the 16S rRNA gene of *Borrelia* spp. by qPCR with CTs ≤ 33. The rodents were captured in the municipalities of Los Córdobas, Urumita and Villa Nueva (Table 2). Subsequent PCR protocols were positive only for the 16S rRNA gene. However, only two of the three nested reactions flanking both extremes of the 16S rRNA gene, but not overlapping, yielded amplicons of expected size (hemi nested A, heminested B). Good quality sequences were generated from samples of two rodents of the genus *Zygodontomys* from the departments of La Guajira and Córdoba (Fig. 1) and corresponded to sequences of 603–673 bp (heminested A) and 344–408 bp (heminested B). The BLASTn analysis of the fourth fragments showed a 98.16–96.02% of identity with *Borrelia* sp. R57 (GenBank accession number AY626138) (Table 2). The alignment for the 16S rRNA gene is constructed with 72 sequences, 70 downloaded from Genbank and two of our own. This alignment was adjusted to the size of the two sequences obtained (603–673 bp), products of the nPCR (hemi nested A). Phylogenetic analysis demonstrated a clustering with *Borrelia* sp. TIS 37 (GenBank accession number MW633074) [17], *Borrelia* R57 (GenBank accession number AY626138) [19], *Borrelia* sp. CA682 (GenBank accession number KF957670), *Borrelia* sp. ALEPB216 (GenBank accession number KF957671), and with *Borrelia* sp. CA684 (GenBank accession number KF957672) [18]. All these borreliae have been grouped in the group proposed as “rodent group” [17] (Fig. 2).

**Table 2:** Rodents positive by qPCR for *Borrelia* spp

Rodent code	Municipality	Department	Longitude	Latitude	Positive species by qPCR	16S rRNA nPCR primer pairs	bp	Percentage of identity with *Borrelia* sp. R57 (GenBank accession number AY626138)	GenBank accession number
R-53	Los Cordobas	Córdoba	76°20 ′1.74"W	8°49 ′53.1"N	*Zygodontomys* sp*.*	FD3_F/16S1_R	673	96.64%	O557427
						16S2_F/T50_R	344	96.09%	O557428
VN-15	Urumita	La Guajira	75° 20′ 664"W	12° 16′ 979"N	*Zygodontomys* sp.	FD3_F/16S1_R	603	96.02%	OR564036
						16S2_F/T50_R	408	98.16%	O557430
VN 024	Villa Nueva	La Guajira	73° 02′ 261"W	10° 35′ 563"N	*Sigmodon alstoni*	–	–	–	–
VN 025	Villa Nueva	La Guajira	73° 02′ 261"W	10° 35′ 563"N	*Sigmodon alstoni*	–	–	–	–

## Discussion

A *Borrelia* sp*.* from the “rodent group” was detected in *Zygodontomys* and *Sigmodon* in the Colombian Caribbean. Rodents are implicated as reservoirs of *Borrelia* species, mainly of the Bbsl group in the USA and Europe, where the bacteria are endemic [10]. However, there are reports where rodents are involved in the ecoepidemiology of the RF group [7]. Furthermore, some studies have detected species of the *Borrelia* genus in rodents that are in a different phylogenetic clade than the Bbsl and RF groups [17–19]. This was first demonstrated in a study conducted in Spain in 2005, where *Borrelia* sp. R57 was detected in rodents *Apodemus sylvaticus*, *Clethrionomys glareolus*, and *Crocidura russula*, with an infection rate of 8.5–12% by PCR targeting the 16S rRNA gene specific for this detected *Borrelia*, with primers designed by the authors. However, attempts to amplify fragments of *5S-23S*, *ospA*, *flaB*, *rpoB*, and *p66* genes were unsuccessful. In the same study, phylogenetic analyses fragments of 16S rRNA and *groEL* genes demonstrated that the sequences are located in a separate clade from RF and Bbsl groups [19]. These results were corroborated by a study conducted in the USA, where it was found that 43.5% of ear biopsies from *R*. *rattus* were positive for *Borrelia* sp. Phylogenetic analysis of the 16S rRNA gene demonstrated that the sequence from this study is related to the Spanish strain (*Borrelia* sp. R57).

Additionally, in 2021, a study in Australia reported a new species of *Borrelia* in tissue samples from nine rodents of the genus *Rattus*. This detection was obtained with the 16S rRNA v3–4 hypervariable region on the Illumina MiSeq, since attempts to amplify and perform Sanger sequencing of the *flaB* gene were unsuccessful. The identity of the *Borrelia* sequences showed that they were most similar to *Borrelia* sp. R57, as well as, phylogenetic analysis of these Australian sequences forms a separate clade, basal to the three main groups currently described. Therefore, the proposal of a new group of *Borrelia* associated with rodents, the “rodent group,” is suggested [17]. Our results support this hypothesis with the phylogenetic grouping of our sequences in the “rodent group” (Fig. 2). However, in all studies, there have been difficulties in amplifying other genes to confirm the taxonomic assignment of borreliae in this group.

In studies that detected *Borrelia* from the “rodent group”, attempts to detect this species in ticks were unsuccessful. Considering that soft and hard ticks are vectors of *Borrelia* species from the Bbsl group and the RF group, it is necessary to conduct studies to determine which tick species play a vectorial role in the transmission of this newly identified group.

In South America, there are few studies of *Borrelia* in rodents. One of the first studies was carried out in Chile by Thomas et al. in 2020, where PCR detected the presence of *Borrelia* of the Bbsl group in 5% (3/53) of blood samples of the rodents *Oligoryzomys longicaudatus* and *Phyllotis xanthopygus* [15].

Recently, in Colombia, *Borrelia* sp. related to the RF group was detected in tissues from the rodents *Coendou rufescens* and *Microryzomys latissimus*; and related to the Bbsl group in *Thomasomys aureus* and *Mus musculus*, with an infection rate of 1.9% for each group [16].

Few studies have detected *Borrelia* from the “rodent group” and difficulty has been generated in carrying out the molecular characterization of the “species” of this newly proposed group. Unfortunately, in our study, obtaining amplifications of the 16S-rRNA gene was only possible. This may be because this gene is highly conserved, and we are probably dealing with an atypical variant of *Borrelia*.

The phylogenetic analysis showed that our sequences clustered with *Borrelia* sp. TIS 37 (GenBank accession number MW633074) detected in *Rattus rattus* in Australia [17], *Borrelia* sp. R57 (GenBank accession number AY626138) detected in *Clethrionomys glareolus* in Spain [19]. (Gil et al. [19]); *Borrelia* sp. CA682 (GenBank accession number KF957670), *Borrelia* sp. ALEPB216 (GenBank accession number KF957671), and *Borrelia* sp. CA684 (GenBank accession number KF957672) detected in *Rattus rattus* in the USA [18]. The trees topology showed that our study sequences are located in a monophyletic group of borreliae associated only with rodents, basal to the RF group (Fig. 2). Unfortunately, it was only possible to obtain amplifications of the 16S rRNA gene and sequences of other genes could not be recovered to confirm the taxonomic assignment of the *Borrelia* detected in this study.

Our study underscores the need for further research that involves the use of multilocus genes, amplicon based whole genome sequencing with high throughput sequencing, and culturing *Borrelia* sp. from rodent tissues. These approaches could provide a more comprehensive understanding of the eco-epidemiology of *Borrelia* sp. in rodents.

## Conclusions

This is the second detection of *Borrelia* sp. in rodents from Colombia and the first detection of *Borrelia* sp. in the “rodent group” in Latin America.

Furthermore, a wide geographical distribution of borreliae of the “rodent group” is demonstrated, with previous records in Australia, Spain, and the USA, and, with our results, in Colombia, being in South America for the first time.

## Supplementary Information

Additional file 1.

## Data Availability

No datasets were generated or analyzed during the current study.

## Abbreviations

Bbsl: *Borrelia burgdorferi* Sensu lato
RF: Borreliae of the relapsing fever group
qPCR: Real-time polymerase chain reaction
nPCR: Nested polymerase chain reaction
