# Supplementary material for: Rodent group borreliae do occur in wild rodents from the Caribbean region of Colombia
Source: Parasit Vectors. 2024 Nov 29;17:494. doi: 10.1186/s13071-024-06560-7 (PMC11606233; doi:10.1186/s13071-024-06560-7)
Supplement: Supplementary file 1 — Additional file 1. [file 13071_2024_6560_MOESM1_ESM.docx]

Additional file 1: Table S1*.*

Tabla S1*.* Rodents captured in the departments of Córdoba and Guajira, Colombia. Tissue Bank, University of Córdoba, Colombia.

| **Rodent code** | **Year of capture** | **Latitude** | **Longitude** | **Department** | **Municipality** | **Genus** | **Species** | **Tissue** |
| --- | --- | --- | --- | --- | --- | --- | --- | --- |
| R-1 | 2011 | 8°44ˋ15.84ˋˋ | 75°58ˋ42.6ˋˋ | Córdoba | Los Córdobas | *Zygodontomys* | sp. | Spleen |
| R-2 | 2011 | 8°44ˋ15.84ˋˋ | 75°58ˋ42.6ˋˋ | Córdoba | Los Córdobas | *Rattus* | *Rattus rattus* | Spleen |
| R-3 | 2011 | 8°44ˋ15.84ˋˋ | 75°58ˋ42.6ˋˋ | Córdoba | Los Córdobas | *Zygodontomys* | sp. | Spleen |
| R-4 | 2011 | 8°44ˋ15.84ˋˋ | 75°58ˋ42.6ˋˋ | Córdoba | Los Córdobas | *Zygodontomys* | sp. | Spleen |
| R-5 | 2011 | 8°44ˋ15.84ˋˋ | 75°58ˋ42.6ˋˋ | Córdoba | Los Córdobas | *Oligoryzomys* | sp. | Spleen |
| R-6 | 2011 | 8°44ˋ15.84ˋˋ | 75°58ˋ42.6ˋˋ | Córdoba | Los Córdobas | *Oligoryzomys* | sp. | Spleen |
| R-7 | 2011 | 8°44ˋ15.84ˋˋ | 75°58ˋ42.6ˋˋ | Córdoba | Los Córdobas | *Rattus* | *Rattus rattus* | Spleen |
| R-8 | 2011 | 8°44ˋ15.84ˋˋ | 75°58ˋ42.6ˋˋ | Córdoba | Los Córdobas | *Rattus* | *Rattus rattus* | Spleen |
| R-9 | 2011 | 8°44ˋ15.84ˋˋ | 75°58ˋ42.6ˋˋ | Córdoba | Los Córdobas | *Rattus* | *Rattus rattus* | Spleen |
| R-10 | 2011 | 8°44ˋ15.84ˋˋ | 75°58ˋ42.6ˋˋ | Córdoba | Los Córdobas | *Zygodontomys* | sp. | Spleen |
| R-11 | 2011 | 8°44ˋ15.84ˋˋ | 75°58ˋ42.6ˋˋ | Córdoba | Los Córdobas | *Rattus* | *Rattus rattus* | Spleen |
| R-12 | 2011 | 8°44ˋ15.84ˋˋ | 75°58ˋ42.6ˋˋ | Córdoba | Los Córdobas | *Rattus* | *Rattus rattus* | Spleen |
| R-14 | 2011 | 8°51ˋ9.96ˋˋ | 76°20ˋ17.76ˋˋ | Córdoba | Los Córdobas | *Zygodontomys* | sp. | Spleen |
| R-15 | 2011 | 8°51ˋ9.96ˋˋ | 76°20ˋ17.76ˋˋ | Córdoba | Los Córdobas | *Rattus* | *Rattus rattus* | Spleen |
| R-16 | 2011 | 8°51ˋ9.96ˋˋ | 76°20ˋ17.76ˋˋ | Córdoba | Los Córdobas | *Rattus* | *Rattus rattus* | Spleen |
| R-17 | 2011 | 8°51ˋ9.96ˋˋ | 76°20ˋ17.76ˋˋ | Córdoba | Los Córdobas | *Zygodontomys* | sp. | Spleen |
| R-18 | 2011 | 8°51ˋ9.96ˋˋ | 76°20ˋ17.76ˋˋ | Córdoba | Los Córdobas | *Zygodontomys* | sp. | Spleen |
| R-19 | 2011 | 8°51ˋ9.96ˋˋ | 76°20ˋ17.76ˋˋ | Córdoba | Los Córdobas | *Rattus* | *Rattus rattus* | Spleen |
| R-20 | 2011 | 8°50ˋ45.9ˋˋ | 76°20ˋ21.78ˋˋ | Córdoba | Los Córdobas | *Oligoryzomys* | sp. | Spleen |
| R-21 | 2011 | 8°50ˋ45.9ˋˋ | 76°20ˋ21.78ˋˋ | Córdoba | Los Córdobas | *Oligoryzomys* | sp. | Spleen |
| R-22 | 2011 | 8°50ˋ45.9ˋˋ | 76°20ˋ21.78ˋˋ | Córdoba | Los Córdobas | *Zygodontomys* | sp. | Spleen |
| R-23 | 2011 | 8°50ˋ45.9ˋˋ | 76°20ˋ21.78ˋˋ | Córdoba | Los Córdobas | *Zygodontomys* | sp. | Spleen |
| R-24 | 2011 | 8°50ˋ45.9ˋˋ | 76°20ˋ21.78ˋˋ | Córdoba | Los Córdobas | *Zygodontomys* | sp. | Spleen |
| R-25 | 2011 | 8°50ˋ43.5ˋˋ | 76°20ˋ43.5ˋˋ | Córdoba | Los Córdobas | *Zygodontomys* | sp. | Spleen |
| R-26 | 2011 | 8°50ˋ45.9ˋˋ | 76°20ˋ21.78ˋˋ | Córdoba | Los Córdobas | *Oligoryzomys* | sp. | Spleen |
| R-27 | 2011 | 8°50ˋ45.9ˋˋ | 76°20ˋ21.78ˋˋ | Córdoba | Los Córdobas | *Zygodontomys* | sp. | Spleen |
| R-28 | 2011 | 8°50ˋ49.14ˋˋ | 76°20ˋ23.16ˋˋ | Córdoba | Los Córdobas | *Rattus* | *Rattus rattus* | Spleen |
| R-29 | 2011 | 8°50ˋ45.9ˋˋ | 76°20ˋ21.78ˋˋ | Córdoba | Los Córdobas | *Zygodontomys* | sp. | Spleen |
| R-30 | 2011 | 8°50ˋ49.14ˋˋ | 76°20ˋ23.16ˋˋ | Córdoba | Los Córdobas | *Rattus* | *Rattus rattus* | Spleen |
| R-31 | 2011 | 8°50ˋ45.9ˋˋ | 76°20ˋ21.78ˋˋ | Córdoba | Los Córdobas | *Zygodontomys* | sp. | Spleen |
| R-32 | 2011 | 8°50ˋ53.64ˋˋ | 76°20ˋ13.26ˋˋ | Córdoba | Los Córdobas | *Rattus* | *Rattus rattus* | Spleen |
| R-33 | 2011 | 8°50ˋ53.64ˋˋ | 76°20ˋ13.26ˋˋ | Córdoba | Los Córdobas | *Rattus* | *Rattus rattus* | Spleen |
| R-34 | 2011 | 8°50ˋ53.64ˋˋ | 76°20ˋ13.26ˋˋ | Córdoba | Los Córdobas | *Rattus* | *Rattus rattus* | Spleen |
| R-35 | 2011 | 8°50ˋ47.64ˋˋ | 76°20ˋ29.76ˋˋ | Córdoba | Los Córdobas | *Rattus* | *Rattus rattus* | Spleen |
| R-36 | 2011 | 8°50ˋ45.9ˋˋ | 76°20ˋ21.78ˋˋ | Córdoba | Los Córdobas | *Zygodontomys* | sp. | Spleen |
| R-37 | 2011 | 8°50ˋ47.64ˋˋ | 76°20ˋ29.76ˋˋ | Córdoba | Los Córdobas | *Rattus* | *Rattus rattus* | Spleen |
| R-38 | 2011 | 8°50ˋ53.64ˋˋ | 76°20ˋ13.26ˋˋ | Córdoba | Los Córdobas | *Rattus* | *Rattus rattus* | Spleen |
| R-39 | 2011 | 8°50ˋ53.64ˋˋ | 76°20ˋ13.26ˋˋ | Córdoba | Los Córdobas | *Rattus* | *Rattus rattus* | Spleen |
| R-40 | 2011 | 8°50ˋ45.9ˋˋ | 76°20ˋ21.78ˋˋ | Córdoba | Los Córdobas | *Rattus* | *Rattus rattus* | Spleen |
| R-41 | 2011 | 8°50ˋ45.9ˋˋ | 76°20ˋ21.78ˋˋ | Córdoba | Los Córdobas | *Rattus* | *Rattus rattus* | Spleen |
| R-42 | 2011 | 8°50ˋ45.9ˋˋ | 76°20ˋ21.78ˋˋ | Córdoba | Los Córdobas | *Heteromys* | sp. | Spleen |
| R-43 | 2011 | 8°50ˋ45.9ˋˋ | 76°20ˋ21.78ˋˋ | Córdoba | Los Córdobas | *Zygodontomys* | sp. | Spleen |
| R-44 | 2011 | 8°50ˋ45.9ˋˋ | 76°20ˋ21.78ˋˋ | Córdoba | Los Córdobas | *Zygodontomys* | sp. | Spleen |
| R-45 | 2011 | 8°50ˋ45.9ˋˋ | 76°20ˋ21.78ˋˋ | Córdoba | Los Córdobas | *Rattus* | *Rattus rattus* | Spleen |
| R-46 | 2011 | 8°49ˋ50.82ˋˋ | 76°20ˋ6.6ˋˋ | Córdoba | Los Córdobas | *Rattus* | *Rattus rattus* | Spleen |
| R-47 | 2011 | 8°49ˋ50.82ˋˋ | 76°20ˋ6.6ˋˋ | Córdoba | Los Córdobas | *Rattus* | *Rattus rattus* | Spleen |
| R-48 | 2011 | 8°49ˋ50.82ˋˋ | 76°20ˋ6.6ˋˋ | Córdoba | Los Córdobas | *Rattus* | *Rattus rattus* | Spleen |
| R-49 | 2011 | 8°50ˋ49.14ˋˋ | 76°20ˋ23.16ˋˋ | Córdoba | Los Córdobas | *Rattus* | *Rattus rattus* | Spleen |
| R-50 | 2011 | 8°49ˋ53.1ˋˋ | 76°20ˋ1.74ˋˋ | Córdoba | Los Córdobas | *Rattus* | *Rattus rattus* | Spleen |
| R-51 | 2011 | 8°49ˋ53.1ˋˋ | 76°20ˋ1.74ˋˋ | Córdoba | Los Córdobas | *Rattus* | *Rattus rattus* | Spleen |
| R-52 | 2011 | 8°49ˋ53.1ˋˋ | 76°20ˋ1.74ˋˋ | Córdoba | Los Córdobas | *Rattus* | *Rattus rattus* | Spleen |
| R-53 | 2011 | 8°49ˋ53.1ˋˋ | 76°20ˋ1.74ˋˋ | Córdoba | Los Córdobas | *Zygodontomys* | sp. | Spleen |
| R-54 | 2011 | 8°49ˋ53.1ˋˋ | 76°20ˋ1.74ˋˋ | Córdoba | Los Córdobas | *Rattus* | *Rattus rattus* | Spleen |
| R-55 | 2011 | 8°49ˋ53.1ˋˋ | 76°20ˋ1.74ˋˋ | Córdoba | Los Córdobas | *Oligoryzomys* | sp. | Spleen |
| R-56 | 2011 | 8°49ˋ53.1ˋˋ | 76°20ˋ1.74ˋˋ | Córdoba | Los Córdobas | *Rattus* | *Rattus rattus* | Spleen |
| R-57 | 2011 | 8°49ˋ53.1ˋˋ | 76°20ˋ1.74ˋˋ | Córdoba | Los Córdobas | *Rattus* | *Rattus rattus* | Spleen |
| R-58 | 2011 | 8°49ˋ53.1ˋˋ | 76°20ˋ1.74ˋˋ | Córdoba | Los Córdobas | *Rattus* | *Rattus rattus* | Spleen |
| R-59 | 2011 | 8°49ˋ53.1ˋˋ | 76°20ˋ1.74ˋˋ | Córdoba | Los Córdobas | *Rattus* | *Rattus rattus* | Spleen |
| R-60 | 2011 | 8°50ˋ45.9ˋˋ | 76°20ˋ21.78ˋˋ | Córdoba | Los Córdobas | *Zygodontomys* | sp. | Spleen |
| R-61 | 2011 | 8°50ˋ45.9ˋˋ | 76°20ˋ21.78ˋˋ | Córdoba | Los Córdobas | *Rattus* | *Rattus rattus* | Spleen |
| R-62 | 2011 | 8°50ˋ45.9ˋˋ | 76°20ˋ21.78ˋˋ | Córdoba | Los Córdobas | *Zygodontomys* | sp. | Spleen |
| R-63 | 2011 | 8°50ˋ45.9ˋˋ | 76°20ˋ21.78ˋˋ | Córdoba | Los Córdobas | *Zygodontomys* | sp. | Spleen |
| R-64 | 2011 | 8°50ˋ49.14ˋˋ | 76°20ˋ22.62ˋˋ | Córdoba | Los Córdobas | *Proechimys* | sp. | Spleen |
| R-65 | 2011 | 8°50ˋ49.14ˋˋ | 76°20ˋ22.62ˋˋ | Córdoba | Los Córdobas | *Rattus* | *Rattus rattus* | Spleen |
| R-66 | 2011 | 8°50ˋ45.9ˋˋ | 76°20ˋ21.78ˋˋ | Córdoba | Los Córdobas | *Zygodontomys* | sp. | Spleen |
| R-67 | 2011 | 8°50ˋ57.24ˋˋ | 76°20ˋ28.56ˋˋ | Córdoba | Los Córdobas | *Oligoryzomys* | sp. | Spleen |
| R-68 | 2011 | 8°50ˋ45.9ˋˋ | 76°20ˋ21.78ˋˋ | Córdoba | Los Córdobas | *Zygodontomys* | sp. | Spleen |
| R-69 | 2011 | 8°50ˋ45.9ˋˋ | 76°20ˋ21.78ˋˋ | Córdoba | Los Córdobas | *Zygodontomys* | sp. | Spleen |
| R-70 | 2011 | 8°50ˋ45.9ˋˋ | 76°20ˋ21.78ˋˋ | Córdoba | Los Córdobas | *Zygodontomys* | sp. | Spleen |
| R-71 | 2011 | 8°50ˋ49.14ˋˋ | 76°20ˋ22.62ˋˋ | Córdoba | Los Córdobas | *Zygodontomys* | sp. | Spleen |
| R-72 | 2011 | 8°50ˋ49.14ˋˋ | 76°20ˋ22.62ˋˋ | Córdoba | Los Córdobas | *Rattus* | *Rattus rattus* | Spleen |
| R-73 | 2011 | 8°51ˋ9.96ˋˋ | 76°20ˋ17.76ˋˋ | Córdoba | Los Córdobas | *Mus* | *Mus musculus* | Spleen |
| R-74 | 2011 | 8°51ˋ9.96ˋˋ | 76°20ˋ17.76ˋˋ | Córdoba | Los Córdobas | *Mus* | *Mus musculus* | Spleen |
| R-75 | 2011 | 8°51ˋ9.96ˋˋ | 76°20ˋ17.76ˋˋ | Córdoba | Los Córdobas | *Heteromys* | sp. | Spleen |
| R-76 | 2011 | 8°51ˋ9.96ˋˋ | 76°20ˋ17.76ˋˋ | Córdoba | Los Córdobas | *Rattus* | *Rattus rattus* | Spleen |
| R-77 | 2011 | 8°51ˋ9.96ˋˋ | 76°20ˋ17.76ˋˋ | Córdoba | Los Córdobas | *Rattus* | *Rattus rattus* | Spleen |
| R-78 | 2011 | 8°50ˋ34.98ˋˋ | 76°20ˋ10.32ˋˋ | Córdoba | Los Córdobas | *Proechimys* | sp. | Spleen |
| R-79 | 2011 | 8°50ˋ34.98ˋˋ | 76°20ˋ10.32ˋˋ | Córdoba | Los Córdobas | *Zygodontomys* | sp. | Spleen |
| R-83 | 2011 | 8°50ˋ34.98ˋˋ | 76°20ˋ10.32ˋˋ | Córdoba | Los Córdobas | *Heteromys* | sp. | Spleen |
| R-84 | 2011 | 8°50ˋ34.98ˋˋ | 76°20ˋ10.32ˋˋ | Córdoba | Los Córdobas | *Rattus* | *Rattus rattus* | Spleen |
| R-85 | 2011 | 8°50ˋ34.98ˋˋ | 76°20ˋ10.32ˋˋ | Córdoba | Los Córdobas | *Rattus* | *Rattus rattus* | Spleen |
| R-86 | 2011 | 8°50ˋ34.98ˋˋ | 76°20ˋ10.32ˋˋ | Córdoba | Los Córdobas | *Mus* | *Mus musculus* | Spleen |
| R-87 | 2011 | 8°50ˋ34.98ˋˋ | 76°20ˋ10.32ˋˋ | Córdoba | Los Córdobas | *Rattus* | *Rattus rattus* | Spleen |
| R-89 | 2011 | 8°50ˋ34.98ˋˋ | 76°20ˋ10.32ˋˋ | Córdoba | Los Córdobas | *Mus* | *Mus musculus* | Spleen |
| R-90 | 2011 | 8°50ˋ34.98ˋˋ | 76°20ˋ10.32ˋˋ | Córdoba | Los Córdobas | *Rattus* | *Rattus rattus* | Spleen |
| R-91 | 2011 | 8°50ˋ34.98ˋˋ | 76°20ˋ10.32ˋˋ | Córdoba | Los Córdobas | *Rattus* | *Rattus rattus* | Spleen |
| R-92 | 2011 | 8°50ˋ34.98ˋˋ | 76°20ˋ10.32ˋˋ | Córdoba | Los Córdobas | *Rattus* | *Rattus rattus* | Spleen |
| R-93 | 2011 | 8°50ˋ34.98ˋˋ | 76°20ˋ10.32ˋˋ | Córdoba | Los Córdobas | *Rattus* | *Rattus rattus* | Spleen |
| R-94 | 2011 | 8°50ˋ34.98ˋˋ | 76°20ˋ10.32ˋˋ | Córdoba | Los Córdobas | *Rattus* | *Rattus rattus* | Spleen |
| R-95 | 2011 | 8°50ˋ34.98ˋˋ | 76°20ˋ10.32ˋˋ | Córdoba | Los Córdobas | *Rattus* | *Rattus rattus* | Spleen |
| R-96 | 2011 | 8°50ˋ34.98ˋˋ | 76°20ˋ10.32ˋˋ | Córdoba | Los Córdobas | *Rattus* | *Rattus rattus* | Spleen |
| R-97 | 2011 | 8°50ˋ34.98ˋˋ | 76°20ˋ10.32ˋˋ | Córdoba | Los Córdobas | *Rattus* | *Rattus rattus* | Spleen |
| VN 001 | 2012 | 10°45ˋ8ˋˋ | 73°8ˋ57ˋˋ | Guajira | Villa Nueva | *Zygodontomys* | sp. | Spleen |
| VN 002 | 2012 | 10°44ˋ24ˋˋ | 73°7ˋ43ˋˋ | Guajira | Villa Nueva | *Zygodontomys* | sp. | Spleen |
| VN 003 | 2012 | 10°44ˋ25ˋˋ | 73°7ˋ40ˋˋ | Guajira | Villa Nueva | *Zygodontomys* | sp. | Spleen |
| VN 005 | 2012 | 10°44ˋ27ˋˋ | 73°7ˋ33ˋˋ | Guajira | Villa Nueva | *Zygodontomys* | sp. | Spleen |
| VN 006 | 2012 | 10°44ˋ35ˋˋ | 73°7ˋ41ˋˋ | Guajira | Villa Nueva | *Zygodontomys* | sp. | Spleen |
| VN 007 | 2012 | 10°44ˋ40ˋˋ | 73°7ˋ53ˋˋ | Guajira | Villa Nueva | *Zygodontomys* | sp. | Spleen |
| VN 008 | 2012 | 10°44ˋ23ˋˋ | 73°6ˋ21ˋˋ | Guajira | Villa Nueva | *Zygodontomys* | sp. | Spleen |
| VN 009 | 2012 | 10°44ˋ23ˋˋ | 73°6ˋ21ˋˋ | Guajira | Villa Nueva | *Zygodontomys* | sp. | Spleen |
| VN 010 | 2012 | 10°44ˋ23ˋˋ | 73°6ˋ21ˋˋ | Guajira | Villa Nueva | *Zygodontomys* | sp. | Spleen |
| VN 012 | 2012 | 12°32ˋ19ˋˋ | 75°31ˋ4ˋˋ | Guajira | Urumita | *Zygodontomys* | sp. | Spleen |
| VN 013 | 2012 | 12°32ˋ19ˋˋ | 75°31ˋ4ˋˋ | Guajira | Urumita | *Zygodontomys* | sp. | Spleen |
| VN 014 | 2012 | 12°32ˋ19ˋˋ | 75°31ˋ4ˋˋ | Guajira | Urumita | *Oligoryzomys* | sp. | Spleen |
| VN 015 | 2012 | 12°32ˋ19ˋˋ | 75°31ˋ4ˋˋ | Guajira | Urumita | *Zygodontomys* | sp. | Spleen |
| VN 016 | 2012 | 12°32ˋ19ˋˋ | 75°31ˋ4ˋˋ | Guajira | Urumita | *Zygodontomys* | sp. | Spleen |
| VN 017 | 2012 | 12°32ˋ19ˋˋ | 75°31ˋ4ˋˋ | Guajira | Urumita | *Oligoryzomys* | sp. | Spleen |
| VN 018 | 2012 | 12°32ˋ19ˋˋ | 75°31ˋ4ˋˋ | Guajira | Urumita | *Zygodontomys* | sp. | Spleen |
| VN 019 | 2012 | 10°44ˋ23ˋˋ | 73°6ˋ21ˋˋ | Guajira | Villa Nueva | *Zygodontomys* | sp. | Spleen |
| VN 020 | 2012 | 10°44ˋ23ˋˋ | 73°6ˋ21ˋˋ | Guajira | Villa Nueva | *Zygodontomys* | sp. | Spleen |
| VN 021 | 2012 | 12°32ˋ19ˋˋ | 75°31ˋ4ˋˋ | Guajira | Villa Nueva | *Zygodontomys* | sp. | Spleen |
| VN 022 | 2012 | 10°44ˋ23ˋˋ | 73°6ˋ21ˋˋ | Guajira | Villa Nueva | *Zygodontomys* | sp. | Spleen |
| VN 023 | 2012 | 10°44ˋ23ˋˋ | 73°6ˋ21ˋˋ | Guajira | Villa Nueva | *Sigmodon* | *Sigmodon alstoni* | Spleen |
| VN 024 | 2012 | 10°44ˋ23ˋˋ | 73°6ˋ21ˋˋ | Guajira | Villa Nueva | *Sigmodon* | *Sigmodon alstoni* | Spleen |
| VN 025 | 2012 | 10°44ˋ23ˋˋ | 73°6ˋ21ˋˋ | Guajira | Villa Nueva | *Sigmodon* | *Sigmodon alstoni* | Spleen |
| COR01 | 2022 | 8°34ˋ7.6ˋˋ | 75°42ˋ52.9ˋˋ | Córdoba | Montería | *Proechimys* | sp. | Spleen |
| COR02 | 2022 | 8°34ˋ7.6ˋˋ | 75°42ˋ52.9ˋˋ | Córdoba | Montería | *Oligoryzomys* | sp. | Spleen |
| COR03 | 2022 | 8°3ˋ48.4ˋˋ | 76°9ˋ34.3ˋˋ | Córdoba | Tierralta | *Oligoryzomys* | sp. | Spleen |
| COR04 | 2022 | 8°3ˋ48.4ˋˋ | 76°9ˋ34.3ˋˋ | Córdoba | Tierralta | *Zygodontomys* | sp. | Spleen |
| COR05 | 2022 | 8°47ˋ27.8ˋˋ | 76°14ˋ27.8ˋˋ | Córdoba | Los Córdobas | *Oligoryzomys* | sp. | Spleen |
| COR06 | 2022 | 8°47ˋ27.8ˋˋ | 76°14ˋ27.8ˋˋ | Córdoba | Los Córdobas | *Rattus* | *Rattus rattus* | Spleen |
| COR07 | 2023 | 9°14ˋ46ˋˋ | 76°7ˋ43ˋˋ | Córdoba | Moñitos | *Proechimys* | sp. | Spleen |
| COR08 | 2023 | 9°14ˋ46ˋˋ | 76°7ˋ43ˋˋ | Córdoba | Moñitos | *Rattus* | *Rattus rattus* | Spleen |
| COR09 | 2023 | 8°34ˋ7.6ˋˋ | 75°42ˋ52.9ˋˋ | Córdoba | Montería | *Oligoryzomys* | sp. | Spleen |
| COR10 | 2023 | 8°34ˋ7.6ˋˋ | 75°42ˋ52.9ˋˋ | Córdoba | Montería | *Oligoryzomys* | sp. | Spleen |
| COR11 | 2023 | 8°53ˋ23ˋˋ | 75°45ˋ31.5ˋˋ | Córdoba | Cereté | *Mus* | *Mus musculus* | Spleen |
| COR12 | 2023 | 8°53ˋ23ˋˋ | 75°45ˋ31.5ˋˋ | Córdoba | Cereté | *Mus* | *Mus musculus* | Spleen |
| COR13 | 2023 | 8°53ˋ23ˋˋ | 75°45ˋ31.5ˋˋ | Córdoba | Cereté | *Mus* | *Mus musculus* | Spleen |
| COR14 | 2023 | 8°53ˋ23ˋˋ | 75°45ˋ31.5ˋˋ | Córdoba | Cereté | *Oligoryzomys* | sp. | Spleen |
| COR15 | 2023 | 8°53ˋ23ˋˋ | 75°45ˋ31.5ˋˋ | Córdoba | Cereté | *Mus* | *Mus musculus* | Spleen |
| COR16 | 2023 | 8°47ˋ27.8ˋˋ | 76°14ˋ27.8ˋˋ | Córdoba | Los Córdobas | *Oligoryzomys* | sp. | Spleen |
| COR17 | 2023 | 8°47ˋ27.8ˋˋ | 76°14ˋ27.8ˋˋ | Córdoba | Los Córdobas | *Oligoryzomys* | sp. | Spleen |
| COR18 | 2023 | 8°47ˋ27.8ˋˋ | 76°14ˋ27.8ˋˋ | Córdoba | Los Córdobas | *Rattus* | *Rattus rattus* | Spleen |
| COR19 | 2023 | 9°15ˋ38.8ˋˋ | 75°48ˋ38.8ˋˋ | Córdoba | Lorica | *Rattus* | *Rattus rattus* | Spleen |
| COR20 | 2023 | 9°15ˋ38.8ˋˋ | 75°48ˋ38.8ˋˋ | Córdoba | Lorica | *Necromys* | sp. | Spleen |
| COR21 | 2023 | 9°14ˋ46ˋˋ | 76°7ˋ43ˋˋ | Córdoba | Moñitos | *Zygodontomys* | sp. | Spleen |
| COR22 | 2023 | 9°14ˋ46ˋˋ | 76°7ˋ43ˋˋ | Córdoba | Moñitos | *Rattus* | *Rattus rattus* | Spleen |
| COR23 | 2023 | 8°47ˋ27.8ˋˋ | 76°14ˋ27.8ˋˋ | Córdoba | Los Córdobas | *Rattus* | *Rattus rattus* | Spleen |
| COR24 | 2023 | 8°34ˋ7.6ˋˋ | 75°42ˋ52.9ˋˋ | Córdoba | Montería | *Proechimys* | sp. | Spleen |
| COR25 | 2023 | 8°34ˋ7.6ˋˋ | 75°42ˋ52.9ˋˋ | Córdoba | Montería | *Rattus* | *Rattus rattus* | Spleen |
| COR26 | 2023 | 8°34ˋ7.6ˋˋ | 75°42ˋ52.9ˋˋ | Córdoba | Montería | *Rattus* | *Rattus rattus* | Spleen |
| COR27 | 2023 | 8°47ˋ27.8ˋˋ | 76°14ˋ27.8ˋˋ | Córdoba | Los Córdobas | *Rattus* | *Rattus rattus* | Spleen |
| COR28 | 2023 | 8°47ˋ27.8ˋˋ | 76°14ˋ27.8ˋˋ | Córdoba | Los Córdobas | *Zygodontomys* | sp. | Spleen |
| COR29 | 2023 | 9°15ˋ38.8ˋˋ | 75°48ˋ38.8ˋˋ | Córdoba | Lorica | *Necromys* | sp. | Spleen |
| COR030 | 2023 | 9°15ˋ38.8ˋˋ | 75°48ˋ38.8ˋˋ | Córdoba | Lorica | *Necromys* | sp. | Spleen |
| COR031 | 2023 | 8°34ˋ7.6ˋˋ | 75°42ˋ52.9ˋˋ | Córdoba | Montería | *Rattus* | *Rattus rattus* | Spleen |
| COR032 | 2023 | 8°34ˋ7.6ˋˋ | 75°42ˋ52.9ˋˋ | Córdoba | Montería | *Rattus* | *Rattus rattus* | Spleen |
| COR033 | 2023 | 8°34ˋ7.6ˋˋ | 75°42ˋ52.9ˋˋ | Córdoba | Montería | *Rattus* | *Rattus rattus* | Spleen |
| COR034 | 2023 | 8°34ˋ7.6ˋˋ | 75°42ˋ52.9ˋˋ | Córdoba | Montería | *Rattus* | *Rattus rattus* | Spleen |
| COR035 | 2023 | 8°34ˋ7.6ˋˋ | 75°42ˋ52.9ˋˋ | Córdoba | Montería | *Rattus* | *Rattus rattus* | Spleen |
| COR036 | 2023 | 8°34ˋ7.6ˋˋ | 75°42ˋ52.9ˋˋ | Córdoba | Montería | *Rattus* | *Rattus rattus* | Spleen |
| COR037 | 2023 | 8°34ˋ7.6ˋˋ | 75°42ˋ52.9ˋˋ | Córdoba | Montería | *Rattus* | *Rattus rattus* | Spleen |
| COR038 | 2023 | 9°14ˋ46ˋˋ | 76°7ˋ43ˋˋ | Córdoba | Moñitos | *Rattus* | *Rattus rattus* | Spleen |
| COR039 | 2023 | 9°14ˋ46ˋˋ | 76°7ˋ43ˋˋ | Córdoba | Moñitos | *Proechimys* | sp. | Spleen |
| COR40 | 2023 | 9°14ˋ46ˋˋ | 76°7ˋ43ˋˋ | Córdoba | Moñitos | *Zygodontomys* | sp. | Spleen |
